# NGF FLIPs TrkA onto the death TRAIL in neuroblastoma cells

**DOI:** 10.1038/cddis.2016.49

**Published:** 2016-03-10

**Authors:** P Ruggeri, L Cappabianca, A R Farina, L Gneo, A R Mackay

**Affiliations:** 1 Department of Applied Clinical and Biotechnological Sciences, University of L'Aquila, L'Aquila, Italy

Spatial and temporal changes in the expression of nerve growth factor (NGF) and its receptor tropomyosin-related kinase A (TrkA) are important in regulating cell fate choice as neuroblast delineate from the neural crest, migrate throughout the developing embryo, differentiate along the sympatho-adrenal lineage and eventually give rise to the sympathetic nervous system (SNS). Within this context, the ligation of active NGF by TrkA protects neuroblasts against apoptosis and promotes differentiation; pro-form NGF, in the absence of TrkA, promotes neuroblast apoptosis via the low-affinity NGF receptor p75^NTR^ and sortilin, and in the absence of NGF, TrkA recruits p75^NTR^ as a hired killer to induce neuroblast apoptosis. This variety of potential pro- and anti-apoptotic outcomes is required to eliminate neuroblast excess yet maintain sufficient neuroblast numbers for development of the fully functional SNS.^1^

Neuroblastomas (NBs) are devastating extra-cranial paediatric tumours that originate from neuroblasts of neural crest origin transformed at different stages along the sympatho-adrenal lineage. Not surprisingly and consistent with this origin, NBs are comprised of cells that are blocked in the particular differentiation state at the time of transformation and selected for resistance to apoptosis by a combination of oncogene activation, oncosuppressor inactivation and physiological apoptosis protection mechanism(s) conserved at the time of transformation, and exhibit different patterns of neurotrophin and neurotrophin receptor expression.^1, 2^ The characterisation of the mechanisms through which NBs resist apoptosis is critical for the future development of novel pro-apoptotic therapeutic strategies for use in this tumour type.

Apoptosis can be activated via extrinsic and/or intrinsic pathways.^3^ The identification of tumour necrosis factor*α* (TNF*α*) as a pro-apoptotic cytokine paved the way for characterisation of the extrinsic apoptosis pathway and led to identification of the TNF*α*, Fas, TNF-related apoptotis-inducing ligand (TRAIL) and Apo3L pro-apoptotic cytokine family. Pro-apoptotic cytokines induce apoptosis through the extrinsic and/or intrinsic pathways by activating cognate TNFR1, FasL, FasR, DR3, DR4 and DR5 death receptors. Among this cytokine family, TRAIL is considered to be a promising chemotherapeutic agent because of its selective pro-apoptotic action on tumour, but not non-transformed cells.^4, 5, 6^ However, the potential therapeutic application of TRAIL in NB has been hampered by observations of TRAIL resistance in NB models, which has been attributed to caspase-8 silencing, altered decoy and functional TRAIL receptor expression, expression of the cellular FLICE-like inhibitory protein (cFLIP) inhibitory analogue of caspase-8, protective IP3K/Akt/mTor signalling or protective RIP/nuclear factor kappa binding (NF-*κ*B) signalling. Accordingly, re-sensitisation of TRAIL-resistant NB cells to TRAIL-induced apoptosis has been reported for agents that increase the expression of caspase-8, ectopic overexpression of functional TRAIL receptors, the inhibition of cFLIP expression and agents that promote a pro-apoptotic expression pattern of TRAIL-induced apoptosis components.^7, 8, 9, 10^

In our recent *Cell Death Discovery* publication,^11^ we describe a novel NGF-mediated mechanism for sensitising TrkA-expressing SH-SY5Y NB cells to TRAIL-induced apoptosis that abrogates anchorage-independent tumorigenic growth *in vitro*. In our study, the TRAIL-resistant phenotype of SH-SY5Y NB cells (Figure 1a) was found not to be dependent upon the loss of expression of any single component of the TRAIL-induced apoptotic pathway, but to be associated with relatively high-level cell surface expression of functional DR4 and DR5 TRAIL receptors, relatively low-level expression of decoy (DcR1 and DcR2) TRAIL receptors and very low-level expression of the anti-apoptotic B-cell lymphoma 2 (Bcl-2) family proteins: Bcl-2, B-cell lymphoma extra large (Bcl-xL) and myeloid cell leukaemia-1 (Mcl-1). A central role for cFLIP in the TRAIL-resistant SH-SY5Y phenotype was confirmed by small interfering RNA (siRNA) cFLIP knockdown, which sensitised SH-SY5Y cells to TRAIL-induced apoptosis in the absence of NGF and by ectopic cFLIP overexpression, which abrogated NGF sensitisation of TrkA-expressing SH-SY5Y cells to TRAIL-induced apoptosis. The mechanism through which NGF-sensitised TrkA-expressing SH-SY5Y cells to TRAIL-induced apoptosis (Figure 1b) did not result, however, from NGF downregulation of cFLIP expression, but rather from cFLIP sequestration by NGF-activated TrkA receptors. This reduced cFLIP recruitment and increased caspase-8 recruitment to TRAIL-activated DR4-containing death receptor complexes, resulting in the cleavage and activation of caspase-8 and subsequent caspase-3-mediated apoptosis. This characterises the activated TrkA receptor as a novel competitive inhibitor of cFLIP recruitment to activated TRAIL receptors and, therefore, a novel regulator of TRAIL-induced apoptosis.

NGF sensitisation of TrkA-expressing SH-SY5Y NB cells to TRAIL-induced apoptosis was abrogated by TrkA tyrosine kinase and caspase inhibitors, associated with permeabilisation of the outer mitochondrial membrane, increasing cytosolic levels of mitochondrial Omi/Htr2a and cytochrome *c*, and reducing cytosolic levels of X-linked inhibitor of apoptosis, and was inhibited by ectopic overexpression of Bcl-xL, confirming that apoptosis was TrkA tyrosine kinase dependent, caspase dependent and mediated through the intrinsic mitochondrial pathway. Furthermore, NGF sensitisation of TrkA-expressing SH-SY5Y NB cells to TRAIL-induced apoptosis was temporary, limited to the first 6 h of NGF treatment and was eventually inhibited by increased Mcl-1 expression, but could be further optimised by siRNA Mcl-1 knockdown and by NF-*κ*B inhibitors.

The discovery of this novel pro-apoptotic TRAIL-mediated immunological dimension to NGF-TrkA function, described in our *Cell death Discovery* study,^11^ not only adds to the potential ways in which NGF interaction with TrkA regulates the cell fate choice of neuroblasts, but may also extend to other TrkA-expressing cell types, other neurotrophin/neurotrophin receptor interactions, and potentially to other members of the TNF family and cognate receptors. We are currently investigating these possibilities. This mechanism may also help to explain the association between expression of fully spliced TrkA receptors, better prognosis and spontaneous remission in NB.^1^ Finally, our observations provide a novel potential pro-apoptotic therapeutic strategy, based on ‘painless' forms of NGF^12^ or alternative TrkA agonists, TRAIL or alternative TRAIL receptor agonists and inhibitors of NF-*κ*B and/or Mcl-1, for future development in the treatment of favourable and unfavourable TrkA-expressing NBs, characterised for the expression of the TRAIL apoptosis pathway components and Bcl-2 family members, and cFLIP-dependent TRAIL resistance.

## Figures and Tables

**Figure 1:**
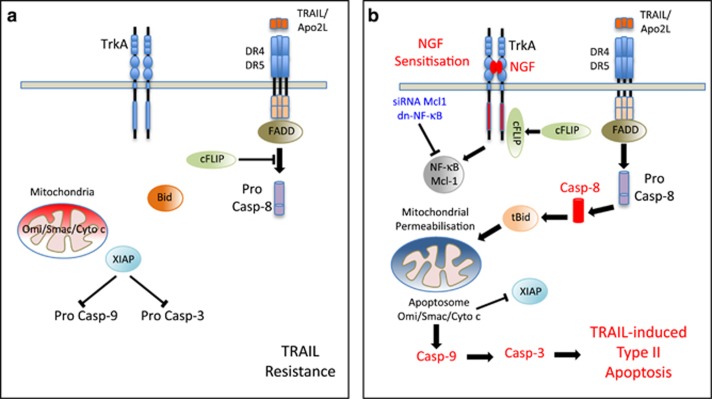
(**a**) Representation of the TRAIL-resistant phenotype exhibited by TrkA SH-SY5Y NB cells in the absence of NGF, demonstrating cFLIP-mediated inhibition of TRAIL-induced pro-caspase-8 activation, absence of Bid cleavage, maintenance of mitochondrial integrity and optimised X-linked inhibitor of apoptosis (XIAP)-mediated blockage of pro-caspase-9 and pro-caspase-3 activation, combining to result in TRAIL resistance. (**b**) Representation of NGF sensitisation of TRAIL-resistant TrkA SH-SY5Y NB cells to TRAIL-induced apoptosis, demonstrating cFLIP sequestration by NGF-activated TrkA, TRAIL-induced activation of pro-caspase-8 and subsequent Bid cleavage (tBid), tBid induction of mitochondrial outer membrane permeabilisation, subsequent release to the cytoplasm of mitochondrial components (Omi/Smac/Cyto *c*), leading to compromised XIAP function, facilitating cytochrome *c*-mediated activation of pro-caspase-9 and pro-caspase-3, resulting in the induction of type II apoptosis through the intrinsic mitochondrial pathway. NGF stimulation of Mcl-1 expression through NF-*κ*B, responsible for the eventual blockage of the temporary NGF-sensitisation response, and relief of this blockage by Mcl-1 siRNA and dominant negative dn-NF-*κ*B are also shown

## Abbreviations

NB: neuroblastoma
TNF: tumour necrosis factor
TRAIL: TNF-related apoptotis-inducing ligand
cFLIP: cellular FLICE-like inhibitory protein
NF-*κ*B: nuclear factor kappa binding
NGF: nerve growth factor
Trk: tropomyosin-related kinase
Mcl-1: myeloid cell leukaemia-1
Bcl-2: B-cell lymphoma 2
Bcl-xL: B-cell lymphoma extra large
BH3-only: Bcl-2 homology domain 3-only
Bid: BH-3 interacting domain death agonist
DcR: decoy receptor
SiRNA: small interfering RNA
